# Constitution and By-Laws of the Society of the Alumni of the Baltimore College of Dental Surgery

**Published:** 1849-01

**Authors:** 


### ARTICLE VII.

### *Constitution and By-Laws of the Society of the Alumni of the Baltimore College of Dental Surgery.*

#### CONSTITUTION.

Art. I. *Object.*—The objects of the Society are the mutual improvement of the graduates and students of the Baltimore College of Dental Surgery, and the advancement of the art and science of dentistry.

Art. II. *Name.*—The Society shall be known and designated as the "Society of the Alumni of the Baltimore College of Dental Surgery.

Art. III. *Officers.*—*Sec.* 1. The officers of the Society shall consist of a President, a Vice-President, a Treasurer, a Corresponding Secretary, a Recording Secretary; also, a Committee on Dental Practice, a Committee on Mechanical Dentistry, a Committee for the selection of subjects for scientific investigation, and a Standing Committee for the purpose of selecting some one of the most distinguished members of the profession, to be invited to deliver the annual address in each succeeding year.

*Sec.* 2. The officers shall be elected by ballot, a majority securing the election of a candidate. All the offices in the Society must be filled by graduate members. And all nominations for any office must be made at the annual meeting preceding the election, except in the case of the officers elected at the first annual meeting.

*Sec.* 3. All officers shall be elected and committees appointed for the term of one year, except the Corresponding Secretary, whose term of office shall continue three years, subject to re-election. The Treasurer, Corresponding Secretary, and Standing Committee shall alone be subject to re-election.

*Sec.* 4. Absent members may vote for the officers of the Society by letter, addressed to the Corresponding Secretary, stating distinctly the name and office of the individual thus voted for, and affixing their signature.

Art. IV. *Membership.*—*Sec.* 1. There shall be three classes of members—graduates, under-graduates and honorary members. The first two shall be the acting members of the Society, after they shall have signed and adopted this constitution, paid into the treasury the initiation fees and dues required by the constitution, and otherwise complied with the by-laws. The honorary members from among such members of the dental and medical professions as shall have in some way distinguished themselves.

*Sec.* 2. All applicants for active membership shall have attended one full course of lectures in the Baltimore College of Dental Surgery.

*Sec.* 3. All active members shall be required, in case of absence from the annual meeting of the Society, to represent themselves by letter, and, at least once in three years, to be present in person, unless excused by vote of the Society.

*Sec.* 4. Any member who shall, after due notice from the Treasurer, fail to pay his dues for three successive years, shall have his name erased from the roll of the Society, unless his dues be remitted by a vote of the Society.

ART. V. *Candidates.—Sec.* 1. All applications for active membership shall be by letter in the handwriting of the applicant, addressed to the Corresponding Secretary, and endorsed by one or more graduate members as vouchers for the character and qualifications of the applicant.

*Sec.* 2. All nominations for honorary membership shall be in writing, signed by three graduate members, and shall be required to state to the Society the claims of the nominee.

*Sec.* 3. Candidates shall be voted for by ball ballot, a majority of three-fourths being necessary for re-election.

*Sec.* 4. All candidates elected shall be required to pay an initiatory fee of three dollars, and two dollars as annual dues for each succeeding year, except those who join the Society at its commencement.

ART. VI.—The name of any member shall be stricken from the roll of the Society, by a vote of three-fourths of the members present at any annual meeting, for conduct deemed at variance with the constitution and spirit of the association.

ART. VII. The annual meeting of the Society shall be held on the day of commencement of the Baltimore College of Dental Surgery, and may be from time to time adjourned, till the business of the year is transacted.

ART. VIII. The funds of the Society shall be devoted to the defraying of contingent expenses, the purchase of scientific apparatus, and the publication of books, tracts, papers and documents, for the advancement of its interest and the furtherance of its objects.

ART. IX. Any revision or amendment of the Constitution may be made at any regular meeting of the Society, by a vote of three-fourths of the members present.

### BY-LAWS.

*Art.* 1. It shall be the duty of the President to preside at all meetings of the Society, and to draw on the Treasurer for all moneys appropriated by the Society.

*Art.* 2. In case of the absence of the President, from sickness or any other cause, it shall be the duty of the Vice-President to preside at all meetings.

*Art.* 3. In the absence of the President and Vice-President, a President *pro tem.* may be appointed at any meeting of the Society.

*Art.* 4. It shall be the duty of the Corresponding Secretary to conduct the correspondence of the Society, agreeably to instructions from the Society, given through the Recording Secretary, or by advice of the President, or, in emergencies, at his own discretion, always observing the spirit of the Constitution. At all elections he shall cast all votes received by letter, according to the instructions of such letters, and shall file away such letters in the archives of the Society. At the opening of the meeting he shall present to the Society all application received for membership. He shall, on every first day of January, notify each member of the precise time appointed for the coming annual meeting. In the absence of the President and Vice-President, it shall be his duty to organize the meeting for the election of a President *pro tem.* He shall be required also to read all reports and dissertations of absent committees and members.

*Art.* 5. It shall be the duty of the Recording Secretary to keep an accurate record of all the proceedings of the Society during his term of office, and to furnish copies of any part or parts thereof, at the order of the President or his substitutes. He shall also keep a roll-book of members' names—which it shall be his duty to call at the opening of each meeting, and to announce the number present to the Society. He shall deliver, at the expiration of his term of office, all books, papers, documents and other articles pertaining to his office, to his successor, or to a committee authorized to receive them.

*Art.* 6. It shall be the duty of the Treasurer to keep all the moneys of the Society; to pay them over to the order of the President, and to keep an accurate account of the same.

*Art.* 7. It shall be the duty of the Committee on Dental Practice to collect the materials and prepare a report on the general practice of the profession during the term of their appointment.

*Art.* 8. It shall be the duty of the Committee on Mechanical Dentistry to receive and examine improvements in, and make reports on the progress of, this branch of dental practice, for the term of their appointment.

*Art.* 9. *Sec.* 1.—It shall be the duty of the Committee for the selection of subjects for scientific investigation to report to the Society, at each annual meeting, *five* distinct subjects, to be disposed of by the Society as hereafter determined.

*Sec.* 2. It shall be the business of the Society, at each annual meeting, to make choice of three from the five subjects presented by the above Committee, and appoint on each subject a committee of three, whose duty it shall be to make report on the same at the next annual meeting.

*Sec.* 3. It shall be the duty of the society to appoint three members, at each meeting, to deliver dissertations at the next annual meeting, on such subjects as they may choose.

*Art.* 10. No member shall speak upon the same question more than twice, or longer than fifteen minutes each time, unless by special permission.

*Art.* 11. Any member may be called to order for indulging, during debate, in any personal recrimination, or for remarks irrelevant to the subject under discussion.

*Art.* 12. The following shall be the order of business to be observed at the annual meetings of the Society:

1. Call to order.
2. Appointment of Committee of Arrangements, consisting of three.
3. Unfinished business.
4. New business and resolutions.
5. Election of new members.
6. Reports of committees.
7. Appointment of committee for the selection of subjects for scientific investigation.
8. Selection of subjects by the Society.
9. Reports of officers.
10. Reports of cases.
11. Discussion of professional subjects.

12. Dissertations, &c.
13. Election of officers and committees.
14. Annual address.
15. Adjournment.

*Art.* 13. The annual address may be delivered immediately after the call to order, at the option of the Society.

*Art.* 14. These By-Laws may be altered or amended at any annual meeting, by a vote of two-thirds of the active members present.

